# Characterization and Quantification of Major Flavonol Glycosides in Ramps (*Allium tricoccum*)

**DOI:** 10.3390/molecules24183281

**Published:** 2019-09-09

**Authors:** Wijdan M. Dabeek, Nik Kovinich, Callee Walsh, Melissa Ventura Marra

**Affiliations:** 1 Division of Animal and Nutritional Sciences, West Virginia University, Morgantown, WV 26506, USA; 2 Division of Plant and Soil Sciences, West Virginia University, Morgantown, WV 26506, USA; 3 Shared Research Facilities, West Virginia University, Morgantown, WV 26506, USA

**Keywords:** ramps, *Allium tricoccum*, flavonols, quercetin, kaempferol

## Abstract

The ramp (*Allium tricoccum*) is a traditional plant in the eastern Appalachian Mountains. Ramps have been used in traditional medicine for their health-promoting roles in lowering blood pressure and cholesterol. Information on the chemical composition of the potentially bioactive components in ramps is limited. Therefore, the aim of this work was to characterize and quantify major flavonols in ramps. Flavonoids were extracted in 50% methanol and 3% acetic acid. Characterization was conducted using UHPLC-PDA-MS and MS/MS, and quantification was performed using UHPLC-PDA detection. The major flavonol glycosides were kaempferol sophoroside glucuronide, quercetin sophoroside glucuronide, kaempferol rutinoside glucuronide, quercetin hexoside glucuronide, quercetin sophoroside, and kaempferol sophoroside. All conjugates were detected in leaves. Quercetin and kaempferol sophoroside glucuronide conjugates were detected in the stem, but no flavonol glycosides were detected in the bulb. The total amounts of the identified quercetin and kaempferol conjugates in whole ramps were 0.5972 ± 0.235 and 0.3792 ± 0.130 mg/g dry weight, respectively. Flavonol conjugates were concentrated in the leaves. To our knowledge, this work is the first to identify and quantify the major flavonol glycosides in ramps. Our findings suggest that specifically the leaves may harbor the potentially bioactive flavonols components of the plant.

## 1. Introduction

*Allium tricoccum* Aiton, commonly known as ramps or wild leeks, is a spring perennial plant belonging to the Liliaceae family. Ramps are native to the deciduous temperate forests in the eastern Appalachian mountains of the United States (U.S) [1]. The scientific name indicates that it is an *allium* vegetable (e.g., onions and garlic) that produces three seeds (*tricoccum*). To germinate, the seeds require a warm and moist season followed by a cold period [2]. Ramps are the first green plants to sprout in rich, moist, shady woods from late March to early May [3]. The mature ramp is a bulb-forming plant with green leaves, white bulbs, and purple or unpigmented stems depending on the variety [4]. There are two varieties of ramps, *A*. *tricoccum var. tricoccum* and *A. tricoccum var. burdickii* [3]. The *triccocum* variety is dominant in the southern Appalachian mountains and has a purple stem and larger leaves than the *burdickii* variety [3]. The *burdickii* variety is more common in the northern mountains and has narrower leaves with an unpigmented stem [3].

Ramps were consumed by the Cherokee First Nations as a spring tonic to replenish nutrients after the winter season [5,6]. The Cherokee First Nations reportedly consumed ramp leaves as a medicinal plant to treat colds and earache [5]. Additionally, traditional folk medicine recommends ramps for lowering blood pressure, lipids, and cholesterol [5,7]. However, the purported health benefits of ramps have not been proven in clinical trials. Studies investigating the health benefits of closely related *allium* vegetables, including wild garlic and leeks, reported blood pressure and cholesterol-lowering effects when fed to rats [8,9]. Flavonoids were reported to be among the bioactive components that elicit the bioactive benefits of *allium* vegetables [10]. Flavonoids are the largest group of plant polyphenols synthesized by the phenylpropanoid pathway [11]. Based on their chemical structure, the variation in the number and distribution of functional groups gives rise to several classes [12]. The most ubiquitous class in plant foods is the flavonol group [13].

The United States Department of Agriculture (USDA) flavonoids database reported that the flavonol class was the only group of flavonoids detected in leeks and spring onions, which are allium vegetables related to ramps [14]. Individual flavonols are unequally distributed in plant parts with different pigments [15]. Flavonols are most commonly found in plants as complex glycoside conjugates that have different sugar moieties linked to an aglycone backbone [16]. The attachment of sugar moieties increases the polarity of flavonols, causing variation in the rates of digestion and absorption in the human body [17]. Although quantifying flavonols in plants is important, a higher content alone does not reflect higher absorption and biological activity in humans [17,18]. Thus, to understand the absorption and bioactivity of flavonols in the human body, it is essential to characterize the glycoside conjugates along with the quantification.

The garlic-like odor of ramps suggests that it contains the volatile sulfur compounds found in *allium* vegetables [19,20]. Calvey et al. identified the sulfur compounds in ramp bulbs harvested from West Virginia and found that the thiosulfinate allicin was the major sulfur component (3 μmol/g dry weight) [20]. Another study reported that ramp’s leaves harvested from southern Ohio and northern Kentucky contain 0.8 mg/g dry weight vitamin C [21]. However, no studies have been published on the characterization and quantification of flavonol glycosides that may contribute to the reported health benefits of ramps. To better understand the potential mechanism of the reported health benefits of ramps, it remains critical that potential bioactive flavonol glycosides be characterized and quantified. Therefore, this study aims to characterize and quantify major flavonol glycosides in whole ramps and the individual parts.

## 2. Results

### 2.1. Optimization of UHPLC Separation of Methanolic Extracts

**Figure 1:**
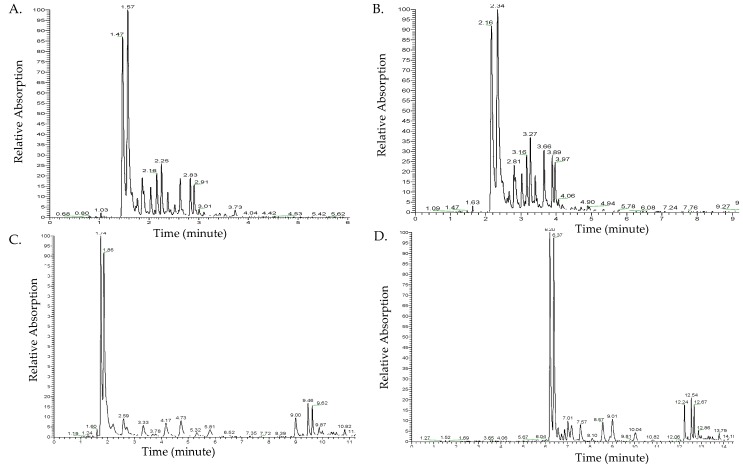
Chromatogram results of different ultra-high-performance liquid chromatography (UHPLC)-photodiode array (PDA) methods examining the flow rate and gradient elution time. Flow rate: (**A**) UHPLC chromatogram of 500 µL/min flow rate, (**B**) UHPLC chromatogram of 300 µL/min flow rate. Gradient elution time: (**C**) UHPLC chromatogram of 21 min gradient elution time, (**D**) UHPLC chromatogram of 24 min gradient elution time.

Figure 1 shows the chromatograms of different ultra-high-performance liquid chromatography (UHPLC)-photodiode array (PDA) methods at 350 nm, including the flow rate and elution times. Peaks were not completely separated at a flow rate of 500 µL/min (Figure 1A). Decreasing the flow rate to 300 µL/min improved the chromatogram separation (Figure 1B). However, the major peak at R_t_ 2.34 min in Figure 1B overlapped with another shoulder peak. Therefore, to achieve a complete separation of peaks, the elution gradient time factor was examined under a flow rate of 300 µL/min.

An increased gradient elution time was achieved by increasing the elution gradient of 90% polar solvent A. This improved the separation of peaks. The elution time of 21 min resulted in a more complete separation and slower elution of the major peaks (Figure 1C) compared to the elution time of 15 min. The two major peaks at R_t_ 1.74 and 1.86 min were not completely separated. An increase in the elution gradient of polar solvent A to 98% with a longer elution time to a total of 24 min resulted in complete separation of all major peaks and increased the retention time (R_t_) of the detected compounds (Figure 1D). To identify flavonol glycosides in ramps, the optimal separation method to be used on UHPLC coupled with photodiode array (PDA) and mass spectrometry (MS) is a flow rate of 300 µL/min and an elution gradient time of 24 min (solvent B gradient: 0 min, 2%; 2 min, 2%; 3 min, 10%; 9 min, 10%; 11 min, 25%; 16 min, 35%; 18 min, 100%; 20 min, 100%; 21 min, 2%; 24 min, 2%).

### 2.2. Characterization of Flavonol Glycosides

**Figure 2:**
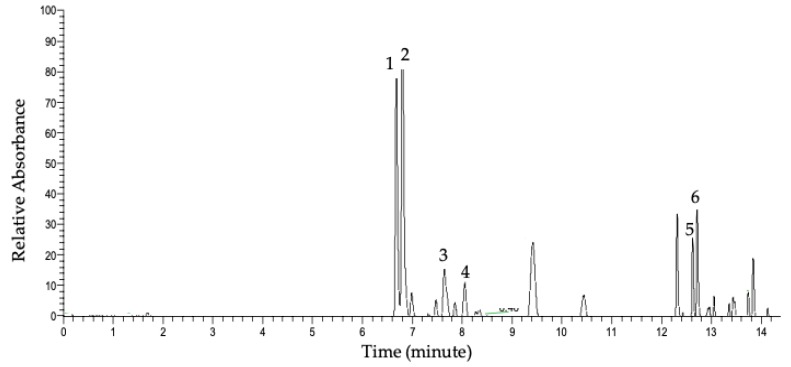
Representation acquired by ultra-high-performance liquid chromatography (UHPLC)-photodiode array (PDA) at 350 nm of whole ramps’ methanolic extract. Peak numbers refer to Table 1, as determined by UHPLC-PDA-MS and MS/MS.

Figure 2 represents the UHPLC-PDA chromatogram of the detected compounds in the methanolic extract at 350 nm. The major detected compounds were identified using data acquired by UHPLC-PDA-MS of the parent ions and data-dependent MS/MS fragmentation. Table 1 shows flavonol glycosides that were identified in whole ramps and organs by UHPLC mass spectrometry (LC-MS) and UHPLC tandem mass spectrometry (LC-MS/MS) using high-resolution and accurate mass measurements. The major flavonol conjugates detected in whole ramps were quercetin and kaempferol glycosides.

Peak 1 detected at R_t_ 6.63 min had a parent mass with an *m*/*z* value of 801.1719. The fragment ion at *m*/*z* 301.0352 indicated the presence of a quercetin backbone after the loss of linked sugar moieties; also observed was the radical anion of quercetin at *m*/*z* 300.0274. The fragment of *m*/*z* 625.1405 is a difference of 176 Da from the parent *m*/*z* value, indicating a loss of a glucuronyl group. Also, the fragment of *m*/*z* 463.0879 indicates the additional loss of a hexosyl group of 162 Da. Based on database searches of the parent ion accurate mass and literature searches, the parent and fragments indicate that the compound was a quercetin sophoroside glucuronide of molecular formula C_33_H_38_O_23_ [22]. Notably, conjugate attachment positions and bond orientations were not able to be determined through LC-MS/MS and will necessitate analysis by nuclear magnetic resonance (NMR) spectroscopy. Additional peaks in the MS/MS spectrum (*m*/*z* 178.9976 and *m*/*z* 343.0454) may indicate a mixed fragmentation spectrum resulting from co-fragmentation of an isobaric species. Peak 2 was detected at R_t_ 6.77 min with a corresponding *m*/*z* 785.1769. The MS/MS fragment ion of *m*/*z* 285. 0246 reflects the kaempferol backbone [M-H-176-324]; furthermore, the fragment peak at *m*/*z* 255 further supports the presence of kaempferol rather than luteolin [22]. The fragment ion at *m*/*z* 609.1459 is a difference of 176 Da from the parent ion, which indicates the loss of a glucuronyl group from the molecule. Based on accurate mass database searches and similar *m*/*z* values reported in the literature, the structure is suggested to be kaempferol sophoroside glucuronide [22]. The additional, prominent fragment peak at *m*/*z* 283 could result from co-fragmentation of isobaric species. Peak 3 with parent *m*/*z* 769.1817 produced the aglycone fragment at *m*/*z* 285.0397, which indicated the kaempferol aglycone backbone; *m*/*z* 284.0324, the radical anion of kampferol, was also observed. Furthermore, a loss of 176 Da from the parent ion, resulting in *m*/*z* 593, indicated the presence of a glucuronyl moiety within the molecule. With these data and accurate mass measurements of the parent molecule, the compound was identified as kaempferol rutinoside glucuronide (C_33_H_38_O_21_) [23]. Peak 4, *m*/*z* 639.1188, was identified as quercetin hexoside glucuronide (C_27_H_28_O_18_) [24], exhibiting the characteristic quercetin aglycone peak of 301, radical anion of *m*/*z* 300, loss of glucuronyl group [M-H-176] resulting in *m*/*z* 463, and further loss of 162 Da to the quercetin aglycone peak [M-H-176-162]. Peak 5 detected at Rt 12.65 min had a parent mass value of *m*/*z* 625.1398 with fragments at *m*/*z* 301.0352 and *m*/*z* 300.0274, indicating the quercetin backbone. The fragment peak at *m*/*z* 463.0890 equates to a 162-Da loss, indicating a loss of a hexosyl group. This molecule was identified as a quercetin sophoroside [25,26].

Similarly, the parent mass of compound 6 with *m*/*z* 609.1443 detected at R_t_ 12.72 showed a fragment at *m*/*z* 285.0405 representing the kaempferol backbone and a fragment of *m*/*z* 446.0850, which is a 162-Da loss from the parent *m*/*z* value, suggesting a loss of one hexosyl group. The fragment peak at *m*/*z* 255 further supports the presence of kaempferol rather than luteolin. The identified conjugate is kaempferol sophoroside [25,27]. The additional, prominent fragment peak at *m*/*z* 283 could result from co-fragmentation of isobaric species.

Supplemental Figures S1–S6 show the MS spectra of the parent *m*/*z* values and fragments of the major flavonol glycosides detected, along with the chemical structures. When separate ramp organs were analyzed, all identified glycosides were detected in leaves. Quercetin and kaempferol sophoroside glucuronide conjugates, however, were detected in the stem. No flavonol glycosides were detected in the bulb.

### 2.3. Quantification of Major Flavonol Glycosides

**Figure 3:**
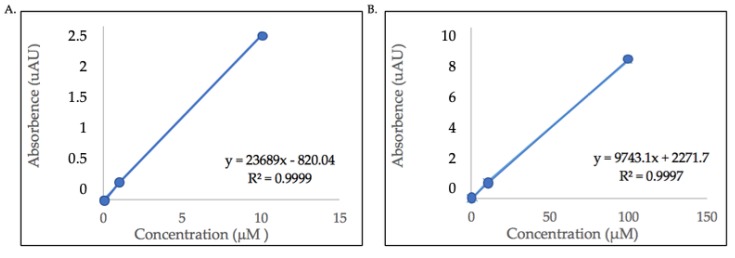
Calibration curves with linear regression lines and equations of (**A**) area under the curve of kaempferol aglycone standard (10, 1, 0.1, 0.01 μM), and (**B**) area under the curve of quercetin aglycone standard (100, 10, 1, 0.1, 0.01 μM). All absorbance values (*Y*-axis) are multiples of 10^5^.

The flavonol content of the detected glycosides were estimated using quercetin and kaempferol aglycone standards because commercial standards for the identified compounds are not commercially available. Calibration curves of quercetin (100, 10, 1, 0.1, 0.01 μM) and kaempferol (10, 1, 0.1, 0.01 μM) standards were plotted with the peak area or absorbance (*Y*-axis) versus standard concentrations (*X*-axis). The linear regression equations were y = 9743.1x + 2271.7, (quercetin, R^2^ = 0.9997) and y = 23689x − 820.04 (kaempferol, R^2^ = 0.9999). Figure 3 shows the calibration curves with linear regression equations of both quercetin and kaempferol aglycones.

Using the calibration curves, absolute amounts of the various conjugates were measured in extracts of the whole ramp, leaf, stem, and bulb. Table 2 shows the quantification of the identified flavonol glycosides and whole ramps and organs. The total amount of the identified quercetin conjugates was highest in leaves, followed by whole and stem (3.582 ± 1.06, 0.5972 ± 0.235, and 0.0112 ± 0.003 mg/g DW, respectively). Similarly, the total amount of identified kaempferol conjugates was highest in leaves followed by the whole plant, and the stem (2.323 ± 0.787, 0.3792 ±0.130, and 0.0079 ± 0.001 mg/g DW, respectively). The quercetin glycosides present in whole ramps extract were quercetin sophoroside glucuronide, sophoroside, and hexoside glucuronide representing 78.8%, 12.2%, and 9.0% of the reported total quantity, respectively. Kaempferol glycosides in whole ramps included sophoroside glucuronide, sophoroside, and rutinoside glucuronide representing 72.9%, 13.2%, and 13.9% of the reported total quantity, respectively. Trace amounts of quercetin sophoroside glucuronide and kaempferol sophoroside glucuronide were detected in the stem.

**Figure 4:**
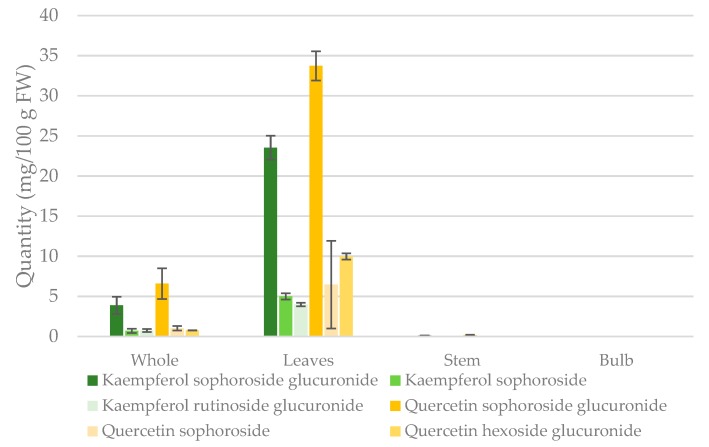
Estimated contents of the major flavonol glycosides identified in whole ramps and separate organs using UHPLC-PDA. Standard deviation bars are shown for *n* = 3. Quantities reported as mg/100g fresh weight (FW).

Ramps are more commonly consumed as fresh plant; one cup of fresh ramps is 100 g. Figure 4 shows quantities of quercetin and kaempferol conjugates presented as mg/100g fresh weight in whole ramps and separate organs. Analysis of the major flavonol glycosides by UHPLC-PDA in individual organs showed that all quercetin and kaempferol conjugates were concentrated in the leaves, with the highest being quercetin sophoroside glucuronide, kaempferol sophoroside glucuronide, quercetin sophoroside glucuronide, quercetin sophoroside, kaempferol sophoroside, and kaempferol rutinoside glucuronide, respectively. The major quercetin and kaempferol conjugates in whole ramps and leaves are quercetin sophoroside glucuronide, and kaempferol sophoroside glucuronide.

## 3. Discussion

This is the first study to characterize and quantify major flavonol glycosides in whole ramps (*Allium tricoccum*) and individual parts. We found that the two major flavonols in ramps were quercetin and kaempferol conjugates. The two glycosides representing more than half of the identified flavonols in ramps were quercetin and kaempferol sophoroside glucuronides. All major quercetin and kaempferol conjugates were concentrated in leaves. Results from UHPLC-PDA-MS and MS/MS is considered tentative and should be confirmed by NMR analysis.

### 3.1. Optimization of UHPLC Separation of Methanolic Extracts

The UHPLC-PDA protocol used to detect flavonol glycosides is specific to the analysis plant, properties of the desired compounds, and the chromatogram column [28,29]. Therefore, the UHPLC-PDA protocol was developed for the detection of flavonol glycosides in ramps. After examining the effect of changing the flow rate, a rate of 300 μL/min improved peaks’ separation and was used to examine the effect of the gradient elution time for complete separation. An increase in the gradient elution time, specifically, polar solvent A, improved the separation of the major peaks because it allowed more time for the detection of polar compounds. Initially, the separation protocol was based on a previously reported method for the detection of anthocyanins [30]. Anthocyanins, however, have a different polarity than flavonol glycosides. The percentage and elution time of the polar solvent A is dependent on the polarity of the desired compounds [31]. In the unknown ramps’ extract, the detected flavonol glycosides are polar and thus modification of the gradient elution time improved the separation of flavonol glycosides in ramps’ methanolic extract.

### 3.2. Characterization of Major Glycosides in Ramps

The UHPLC-PDA method developed for the detection of flavonols was used to characterize the major flavonol glycosides in ramps. In this study, we found that the major flavonol backbones in ramps were quercetin and kaempferol, which is consistent with the flavonol content reported in the USDA flavonoid database in similar *allium* vegetables, spring onions and leeks [14]. Leeks also contained the flavonol myricetin, which was not detected in ramps [14]. The forms of quercetin and kaempferol conjugates that contributed to more than half of the identified flavonols were identified to be kaempferol sophoroside glucuronide and quercetin sophoroside glucuronide based on accurate mass and MS/MS data. These compounds were detected in onion (*Allium cepa*) [24,32] but as minor glycosides. Although analysis of another closely related *allium* plant, wild garlic, showed the presence of kaempferol conjugates, the sugar moieties linked to the kaempferol backbone were different than in ramps [33]. Kaempferol in wild garlic was found to be mainly linked to the disaccharide neohesperidoside [33]. The characterization of quercetin and kaempferol sophoroside conjugates represent the intermediate compounds for the synthesis of the major conjugates, sophoroside glucuronide. Quercetin and kaempferol sophorosides were mainly identified in broccoli (*Brassica oleracea*) [34].

The characterization of flavonol conjugates in ramps is important in understanding the digestion and absorption of the conjugates and thus potential bioactivity in humans. Unconjugated flavonol forms are non-polar and thus are passively absorbed across the small intestine into the blood [35]. The attachment of sugar moieties in plants, however, increases polarity and therefore impacts the absorption into the blood [18]. Clinical trials examining the extent of digestion and absorption of flavonol conjugates similar to the glycosides in ramps are limited. It was reported that quercetin conjugates in onion are rapidly digested and absorbed [36]. However, the major quercetin glycosides characterized in onion are -3,4′-diglucoside and -4′-glucoside [37]. Conversely, quercetin rutinoside detected in black tea was poorly absorbed into the blood [38]. The difference in the extent of digestion and absorption is dependent on the type of sugar linked to quercetin and the kaempferol backbone [39,40]. The attachment of glucose moiety in onions allowed rapid absorption by an active transporter into the blood [41]. Attachment of rutinoside conjugate, however, decreases absorption into the blood due to a lack of the transporter [42]. Also, a clinical trial investigated the extent of the digestion and absorption of kaempferol sophoroside in broccoli and results showed mild absorption into the blood [43]. However, kaempferol glucuronide conjugates detected in endive were rapidly absorbed [44]. Clinical trials are needed to investigate the extent of digestion and absorption of the major flavonol glycosides detected in ramps.

In this study, all identified conjugates were detected in ramps’ leaves. Kaempferol conjugates, but not quercetin, were detected mainly in wild garlic’s leaves [33]. Although quercetin was found to be concentrated in the onion bulb [15], no flavonol glycosides were detected in the ramps’ bulb. Previous reports, however, found that ramps’ bulb contains the sulfur compounds thiosulfinates, mainly allicin [20]. These compounds were also detected in garlic, which provides cardiovascular-protective benefits, including a bloodpressure-lowering effect [45,46]. Thus, the potential health benefits of ramps reported in folk medicine could be related to a synergistic effect between the flavonols in leaves and stem and sulfur compounds in bulbs. Clinical studies investigating the potential health benefits and effective dosage of ramps are needed.

### 3.3. Quantification of Major Flavonol Glycosides

Estimated total amounts of the major identified flavonol glycosides in this study indicated that the consumption of one cup (100 g) of fresh whole ramps provides 8.361 and 5.310 mg of the major quercetin and kaempferol glycosides, respectively. These amounts are higher than the total content of quercetin and kaempferol reported in leeks (0.9 and 2.67 mg/100 g FW) [14]. Spring onion, however, contained higher levels of total quercetin but not kaempferol (12.6 and 4.1 mg/100g FW, respectively) [14]. Notably, the quantities reported in this study represented total amounts of the major identified glycosides instead of the absolute flavonol content. Thus, the actual absolute quantities of quercetin and kaempferol in ramps might be slightly higher.

Flavonol glycosides in ramp organs are concentrated in leaves. Consumption of one cup of fresh ramps leaves provides 50.15 and 32.51 mg of quercetin and kaempferol glycosides, respectively. The content of flavonol glycosides in wild garlic leaves was higher than ramps leaves (18.5 versus 1.68 mg/g DW, respectively) [47]. Flavonol quantities in wild garlic leaves represented the content of all flavonol glycosides, which explains the difference in quantities between ramps and wild garlic leaves. Epidemiological studies reported that consumption of 15 to 39 mg/day flavonols is negatively associated with cardiovascular disease, such as heart failure [48,49]. Consumption of one cup of fresh ramps leaves provides higher amounts than the potentially effective flavonol dose reported in epidemiological studies. In this study, quercetin conjugates were 21% higher than kaempferol in leaves. This was consistent with a study that quantified total hydrolyzed flavonols in the leaf of red spring onion and found greater quercetin content than kaempferol [15].

## 4. Conclusions

This work marks the first qualitative and quantitative analysis of flavonol glycosides in ramps. The main findings revealed that quercetin and kaempferol sophoroside glucuronides were the major flavonol conjugates in ramps. The detected flavonols were found almost exclusively in the leaves of the plant. These flavonol conjugates provide a potential rationale for the reported health benefits of ramps in folk medicine. Also, quantities of the detected flavonol glycosides provide the information needed to conduct bioavailability studies examining the extent of digestion and absorption. Due to the lack of flavonols in the bulb, it is important to investigate whether any potential reported health benefits of ramps are associated with the consumption of flavonols from leaves, sulfur compounds from the bulb, or a synergistic effect of both flavonols and sulfur compounds from whole ramps. Thus, clinical trials evaluating the potential medicinal benefits of ramps are needed. 

## 5. Materials and Methods

### 5.1. Chemicals

Extraction solvents, including methanol and acetic acid, were purchased from Fisher Scientific (San Jose, CA., USA). Hydrochloric acid, kaempferol (>90% purity), and quercetin aglycone standards were purchased from Sigma Aldrich (St. Louis, MO., USA). All solvents for ultra-high-performance liquid chromatography-photodiode array (UHPLC-PDA) and mass spectrometry (LC-MS/MS) were purchased from Fisher Scientific (USA).

### 5.2. Plant Material

Ramps were harvested as a collection of wild type (variety tricoccum) from Randolph and Pendleton Counties in West Virginia from late April to arly May of 2019. The ramps were cleaned and stored in a −20 °C freezer until used for analysis. The identity of the plants was confirmed by Dr. Z. Fowler, Department of Biology and Core Arboretum Director, West Virginia University. Ramps samples were lyophilized and pulverized for analysis because it was reported that this technique maximizes flavonols’ extraction [50]. Whole ramps were lyophilized (BenchTop Pro, SP Scientific, PA, USA) for six days at −46 °C. A portion of the lyophilized ramps was separated into the bulb, stem, and leaves by cutting the middle purple stem from both ends. The second portion of lyophilized ramps was kept whole. The lyophilized individual parts and whole ramp samples were pulverized separately. The powder of each ramp part and whole ramps were packaged in a small transparent ziplock bags and stored at −80 °C.

### 5.3. Extraction of Flavonol Glycosides

To extract flavonol glycosides, each sample was prepared by homogenizing 5 mg of the pulverized whole ramps or individual parts in 100 μL of a solution containing 50% methanol and 3% acetic acid in distilled water (*v*/*v*). The prepared sample was wrapped with aluminum foil to prevent exposure to light and mixed overnight (~18 h) at room temperature. Extracted samples were centrifuged for 3 min (13.3 g, ThermoScientific, CA, USA). The supernatant was transferred to micro-filters and centrifuged for another 3 min (13.3 g, ThermoScientific, CA, USA). The filtered solvent was diluted with 200 μL of a solution containing 3% acetic acid in distilled water (*v*/*v*) and the resulting solution was transferred to a UHPLC test vial and stored in −80 °C until used for analysis.

### 5.4. Flavonol Glycoside Separation

To identify flavonol glycosides in ramps, a separation protocol first had to be developed. UHPLC-PDA was conducted using an Accela system (Thermo Scientific, San Jose, CA, USA) consisting of an Accela 1250 pump, open autosampler, and PDA detector. Flavonol chromatographic separation was performed on a Luna Omega 1.6 μm Polar C18 100 Å LC column with dimensions of 150 × 2.5 mm (Phenomenex, CA, USA) at 35 °C. The flow rates of 300 and 500 µL/min were tested. Gradient elution rates of a mobile phase for solvent A [5% formic acid in distilled water (*v*/*v*)], and solvent B [5% formic acid in acetonitrile (*v*/*v*)] were adjusted as shown in Table 3. Mobile phase solvents were selected based on a previously reported method [51]. An injection volume of 5 µL was held consistent throughout different methods. Flavonols were detected under a wavelength of 350 nm.

### 5.5. Characterization of Flavonol Glycosides by UHPLC-PDA-MS

To characterize flavonol glycosides, UHPLC-PDA-MS and MS/MS were conducted using an Accela system (Thermo Scientific, San Jose, CA, USA) consisting of Accela 1250 pump, open autosampler, and PDA detector connected to a Q-Exactive–Orbitrap mass spectrometer containing a heated electrospray ionization source (HESI). Flavonol chromatographic separation was performed on a Luna Omega 1.6 μm Polar C18 100 Å LC column with dimensions of 150 × 2.5 mm (Phenomenex, CA, USA) at 35 °C. The optimal chromatographic conditions based on the UHPLC-PDA protocol development results were gradient elution with a mobile phase, solvent A; 5% formic acid in LC-MS grade water (*v*/*v*), and solvent B; 5% formic acid in acetonitrile (*v*/*v*). The gradient was programmed as follows: 0 min, 2% B; 3 min, 10% B; 11 min, 25% B; 16 min, 35% B; 18 min, 100% B; 21 min, 2% B; 24 min, 0% B. For column wash, solvent C, 60% acetonitrile was used for 10 min. The flow rate was 300 µL/min throughout the gradient with an injection volume of 5 µL.

MS analysis was initially performed in full MS mode in both positive and negative polarity modes (positive ion mode data not shown). More efficient detection was observed under the negative mode; thus, all further analysis was conducted under negative polarity mode. Full MS properties were: Resolution 70,000, AGC target 3e6, maximum IT 100 ms, and scan range 200–2000 *m*/*z*. Compounds were identified using LC-MS/MS using data-dependent ion selection to detect the fragments of the major parent peaks. Tentative conjugates were identified by comparing the detected *m*/*z* values within 10 ppm to similar values using Metabolite and Chemical Entity Database (METLIN), Human Metabolome Database (HMDB), and published data. The properties of data-dependent MS/MS fragmentation were: Resolution 35,000, AGC target 2e5, maximum IT 50 ms, loop count 5, (N)CE 33, and charge exclusion of uncharged and ions with three or more charges. HESI source properties were: Sheath gas flow rate of 30, auxillary gas flow rate of 10, spray voltage of 3.6 kV, and capillary temperature of 350 °C. Potential chemical structures detected by UHPLC-PDA-MS based on *m*/*z* values reported in the literature were constructed in ChemDraw Prime 16.0.

Flavonols were detected at the 350-nm wavelength. All chromatogram data were collected and analyzed using Xcalibur software (Thermo Scientific, San Jose, CA, USA).

### 5.6. Quantification of Flavonol Glycosides

UHPLC grade quercetin and kaempferol aglycones were used as external standards. Kaempferol and quercetin stock solutions were prepared in 50% methanol. For the calibration curves, serial dilution was performed to obtain concentrations of 10, 1, 0.1, and 0.01 μM of kaempferol standard and 100, 10, 1, 0.1, and 0.01 μM of quercetin standard. Calibration curves of quercetin and kaempferol aglycones were created using the area under the curve detected by UHPLC-PDA.

To quantify the major flavonol glycosides, the whole ramps’ methanolic extract and individual organs were analyzed by UHPLC-PDA using the same protocol conducted for glycoside detection. Similar to previous quantification methods [52], the contents of quercetin and kaempferol conjugates were estimated based upon their aglycone equivalents because the detection of the conjugates on UHPLC-PDA corresponds to the absorbance of the flavonol backbone under 350 nm. Quantities were converted to mg/g dry weight of plant tissues using the molecular weight of the identified conjugates. Values are reported as means ± standard deviation (SD) for triplicates.

## Figures and Tables

**Table 1:** UHPLC-PDA-MS and MS/MS data and putative identification of flavonol glycosides detected in ramps.

Peak	R_t_ (minute)	[M-H]^-^ (*m/z*)	MS/MS (*m/z*)	Molecular Weight (g/mol)	Molecular Formula	Compound	Parts	Ref *
1	6.63	801.1719	301.0352 625.1405 463.0879 300.0274 343.0454 178.9976	802	C_33_H_38_O_23_	Quercetin sophoroside glucuronide	Leaf, stem	[22]
2	6.77	785.1769	285.0405 446.0851 609.1459 447.0921 283.0246 255.0296 113.0231 300.0271 489.1038	786	C_33_H_38_O_22_	Kaempferol sophoroside glucuronide	Leaf, stem	[22]
3	7.68	769.1817	285.0397 284.0324 593.1509	770	C_33_H_38_O_21_	Kaempferol rutinoside glucuronide	Leaf	[23]
4	8.02	639.1188	301.0344 463.0872 300.0274 271.0247	640	C_27_H_28_O_18_	Quercetin hexoside glucuronide	Leaf	[24]
5	12.65	625.1398	301.0352 300.0274 463.0890 151.0030 178.9976 276.0706	626	C_27_H_30_O_17_	Quercetin-sophoroside	Leaf	[25,26]
6	12.72	609.1443	285.0404 283.0246 446.0842 255.0296 489.1040	610	C_27_H_30_O_16_	Kaempferol-sophoroside	Leaf	[25,27]

* All conjugates were reported in Metabolite and Chemical Entity Database (METLIN), and Human Metabolome Database (HMDB). Abbreviations: R_t_, retention time; *m*/*z*, mass to charge ratio; MS/MS, tandem mass spectrometry fragmentation; ref, references.

**Table 2:** Estimated quantities of the the major identified flavonol glycosides in whole ramps and parts using ultra-high-performance liquid chromatography (UHPLC)-photodiode array (PDA) detection.

Compound	Whole (mg/g)	Leaves (mg/g)	Stem (mg/g)	Bulb (mg/g)
Quercetin	0.5972 ± 0.235	3.582 ± 1.06	0.0112 ± 0.003	-
-sophoroside glucuronide	0.4706 ± 0.137	2.408 ± 0.130	0.0112 ± 0.003	-
-sophoroside	0.0729 ± 0.032	0.4611 ± 0.046	-	-
- hexoside glucuronide	0.0536 ± 0.020	0.7132 ± 0.390	-	-
Kaempferol	0.3792 ± 0.130	2.323 ± 0.787	0.0079 ± 0.001	-
- sophoroside glucuronide	0.2763 ± 0.077	1.682 ± 0.106	0.0079 ± 0.001	-
-sophoroside	0.0503 ± 0.019	0.3565 ± 0.028	-	-
- rutinoside glucuronide	0.0526 ± 0.013	0.2840 ± 0.016	-	-

Quantities reported as mean ± standard deviation (SD) of dry weight (DW), *n* = 3.

**Table 3:** Mobile phase gradient elution of different flow rates and gradient elution times.

Method	Solvent A	Solvent B
**Flow Rate (µL/min)**		
300 500	0 min, 90%; 2 min, 75%; 7 min, 65%; 9 min, 0%; 11 min, 0%; 12 min, 90%; 15 min, 90%	0 min, 10%; 2 min, 25%; 7 min, 35%; 9 min, 100%; 11 min, 100%; 12 min, 10%; 15 min, 10%

**Gradient elution time (minutes)**		
15	0 min, 90%; 2 min, 75%; 7 min, 65%; 9 min, 0%; 11 min, 0%; 12 min, 90%; 15 min, 90%	0 min, 10%; 2 min, 25%; 7 min, 35%; 9 min, 100%; 11 min, 100%; 12 min, 10%; 15 min, 10%
21	0 min, 90%; 6 min, 90%; 8 min, 75%; 13 min, 65%; 15 min, 0%; 17 min, 0%; 18 min, 90%; 21 min, 90%	0 min, 10%; 6 min, 10%; 8 min, 25%; 13 min, 35%; 15 min, 100%; 17 min, 100%; 18 min, 10%; 21 min, 10%
24	0 min, 98%; 2 min, 98%; 3 min, 90%; 9 min, 90%; 11 min, 75%; 16 min, 65%; 18 min, 0%; 20 min, 0%; 21 min, 98%; 24 min, 98%	0 min, 2%; 2 min, 2%; 3 min, 10%; 9 min, 10%; 11 min, 25%; 16 min, 35%; 18 min, 100%; 20 min, 100%; 21 min, 2%; 24 min, 2%

## Supplementary Materials

The following are available online at https://www.mdpi.com/1420-3049/24/18/3281/s1, Figures S1–S6: A) Full scan mass spectrometry (MS) spectrum corresponding to Rt of the chromatographic peaks. B) LC-MS/MS spectrum corresponding to fragmentation of flavonol glycosides. Fragmentation patterns are indicated. Linkages of sugars to the aglycone backbone have not been determined, and only one orientation of the sugar linkages is shown for illustrative purposes.
