# Supplementary material for: Characterization and Quantification of Major Flavonol Glycosides in Ramps (Allium tricoccum)
Source: Molecules. 2019 Sep 9;24(18):3281. doi: 10.3390/molecules24183281 (PMC6767245; doi:10.3390/molecules24183281)
Supplement: Supplementary file 1 [file molecules-24-03281-s001.pdf]

molecules

Characterization and Quantification of Major Flavonol Glycosides in Ramps (*Allium tricoccum*)

Wijdan M. Dabeek [1], Nik Kovinich [2,†], Callee Walsh [3] and Melissa Ventura Marra [1,*]

1 Division of Animal and Nutritional Sciences, West Virginia University, Morgantown, WV 26506, USA; wmdabeek@mix.wvu.edu (W.M.D.)

2 Division of Plant and Soil Sciences, West Virginia University, Morgantown, WV 26506, USA; nikola.kovinich@mail.wvu.edu

3 Shared Research Facilities; West Virginia University, Morgantown, WV 26506, USA;callee.walsh@mail.wvu.edu

† Current Address: Biology Department, York University, Toronto, ON M3J 1P3, Canada; kovinich@yorku.ca

* Correspondence: melissa.marra@mail.wvu.edu; Tel.: +1-304-293-2690

List of figures

Figure S1 A) Full scan mass spectrometry (MS) spectrum corresponding chromatographic peak at Rt 6.63 minutes. B) LC-MS/MS fragmentation spectrum corresponding to of m/z 801.1719, a quercetin sophoroside glucuronide. Fragmentation patterns are indicated. Linkages of sugars to the aglycone backbone have not been determined, and only one orientation of the sugar linkages is shown for illustrative purposes.

Figure S2 A) Full scan mass spectrometry (MS) spectrum corresponding to chromatographic peak atRt 6.77 minutes. B) LC-MS/MS fragmentation spectrum of m/z 785.1769, a kaempferol sophoroside glucuronide Fragmentation patterns are indicated. Linkages of sugars to the aglycone backbone have not been determined, and only one orientation of the sugar linkages is shown for illustrative purposes.

Figure S3 A) Full scan mass spectrometry (MS) spectrum corresponding to Rt 7,68 minutes. B) LC-MS/MS spectrum corresponding to fragmentation of m/z 769.1817, a kaempferol rutinoside lglucuronide . Fragmentation patterns are indicated. Linkages of sugars to the aglycone backbone have not been determined, and only one orientation of the sugar linkages is shown for illustrative purposes.

Figure S4 A) Full scan mass spectrometry (MS) spectrum corresponding to Rt 8.02 minutes. B) LC-MS/MS spectrum corresponding to fragmentation of m/z 639.1188, a quercetin hexoside glucuronide. Fragmentation patterns are indicated. Linkages of sugars to the aglycone backbone have not been determined, and only one orientation of the sugar linkages is shown for illustrative purposes.

Figure S5 A) Full scan mass spectrometry (MS) spectrum corresponding chromatographic peak at Rt 12.65 minutes. B) LC-MS/MS fragmentation spectrum for m/z 625.1398, a quercetin sophoroside. Fragmentation patterns are indicated. Linkages of sugars to the aglycone backbone have not been determined, and only one orientation of the sugar linkages is shown for illustrative purposes.

Figure S6 A) Full scan mass spectrometry (MS) spectrum corresponding chromatographic peak at Rt 12.72 minutes. B) LC-MS/MS fragmentation spectrum corresponding m/z 609.1443, a kaempferol sophoroside. Fragmentation patterns are indicated. Linkages of sugars to the aglycone backbone have not been determined, and only one orientation of the sugar linkages is shown for illustrative purposes.

molecules

MDPI

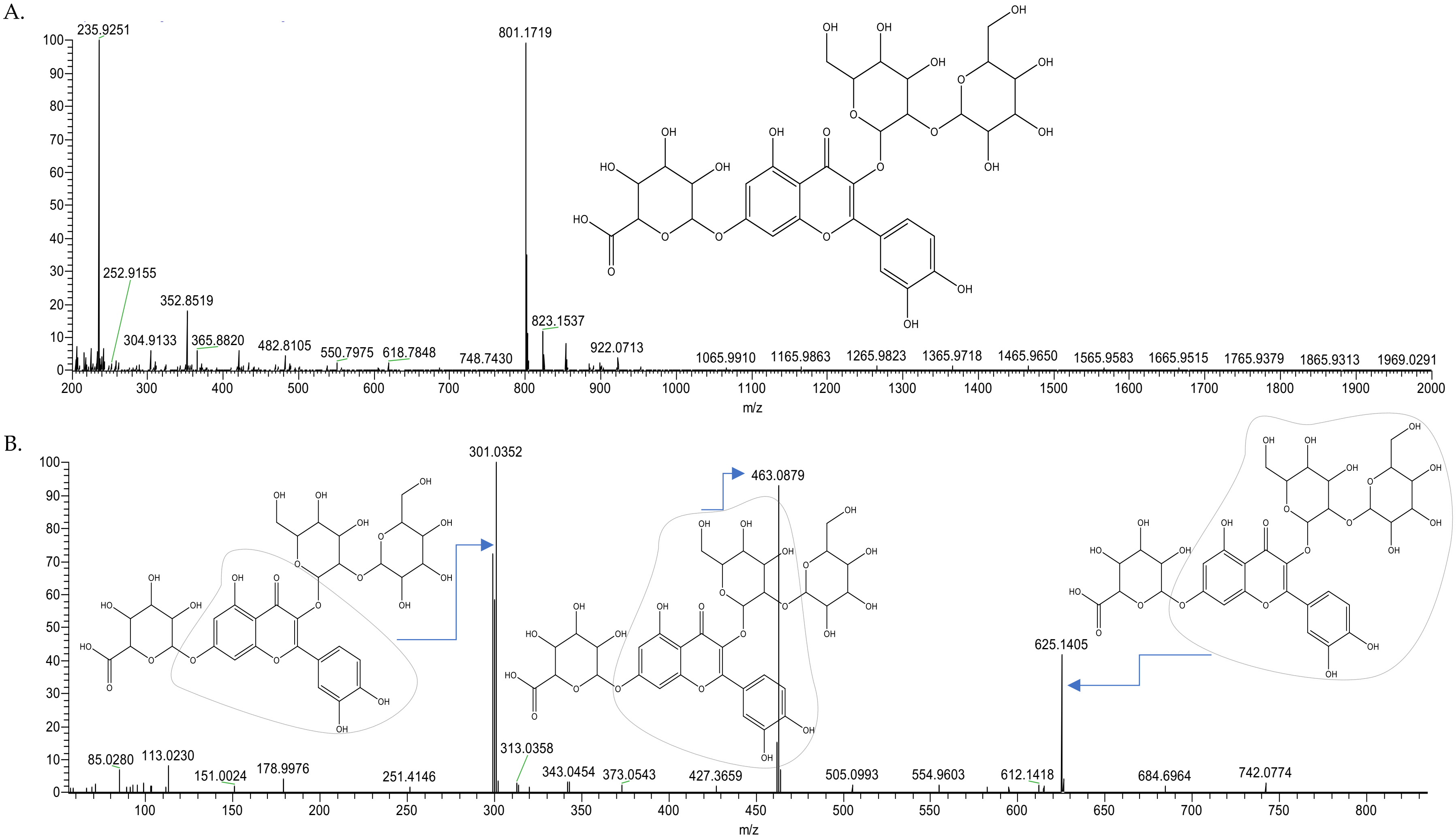

Figure S1 A) Full scan mass spectrometry (MS) spectrum corresponding chromatographic peak at R_t 6.63 minutes. B) LC-MS/MS fragmentation spectrum corresponding to of m/z 801.1719, a quercetin sophoroside glucuronide. Fragmentation patterns are indicated. Linkages of sugars to the aglycone backbone have not been determined, and only one orientation of the sugar linkages is shown for illustrative purposes.

molecules

MDPI

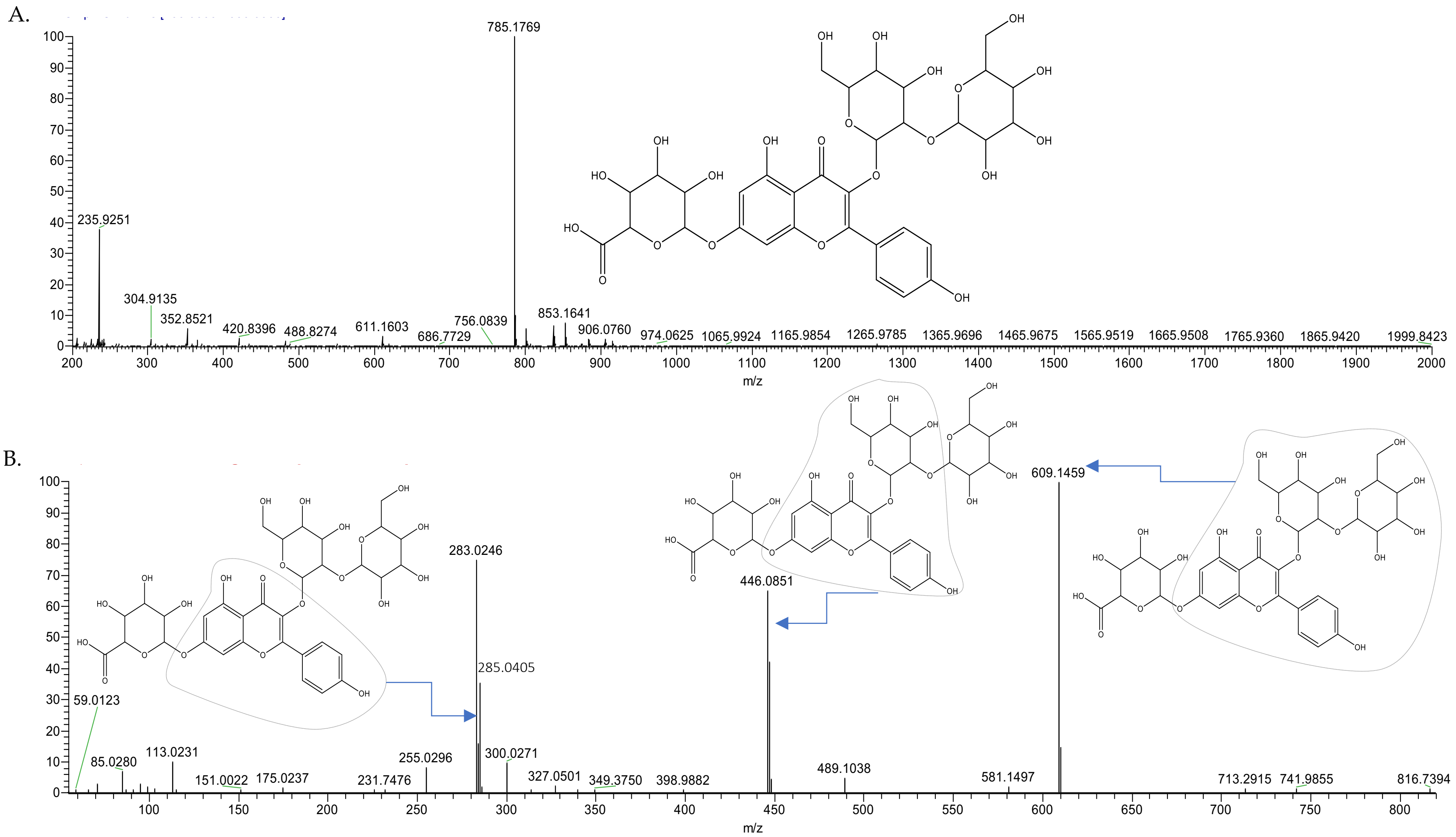

Figure S2 A) Full scan mass spectrometry (MS) spectrum corresponding to chromatographic peak at R_t 6.77 minutes. B) LC-MS/MS fragmentation spectrum of m/z 785.1769, a kaempferol sophoroside glucuronide Fragmentation patterns are indicated. Linkages of sugars to the aglycone backbone have not been determined, and only one orientation of the sugar linkages is shown for illustrative purposes.

molecules
MDPI

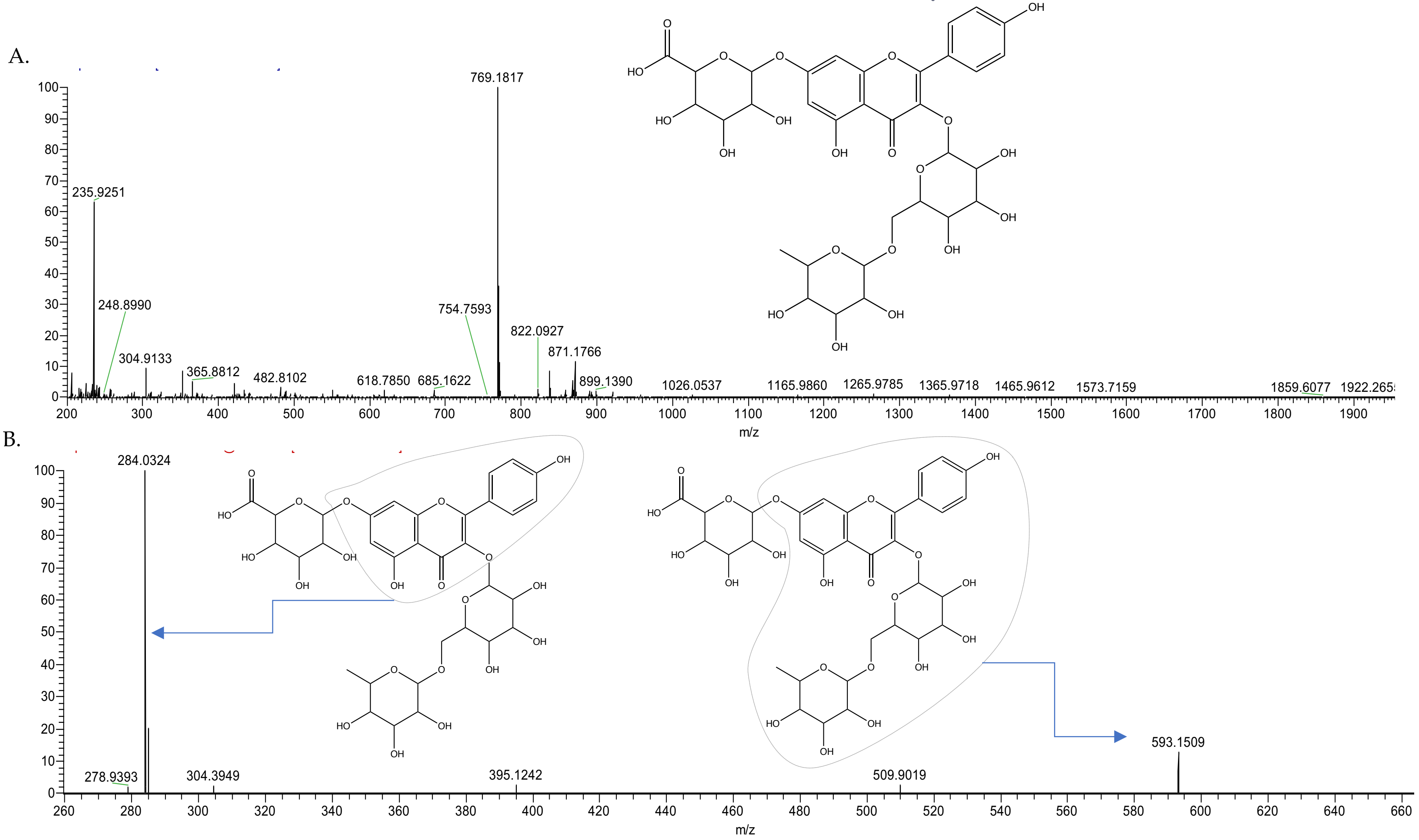

Figure S3 A) Full scan mass spectrometry (MS) spectrum corresponding to R_t 7,68 minutes. B) LC-MS/MS spectrum corresponding to fragmentation of m/z 769.1817, a kaempferol rutinoside lglucuronide. Fragmentation patterns are indicated. Linkages of sugars to the aglycone backbone have not been determined, and only one orientation of the sugar linkages is shown for illustrative purposes.

molecules

MDPI

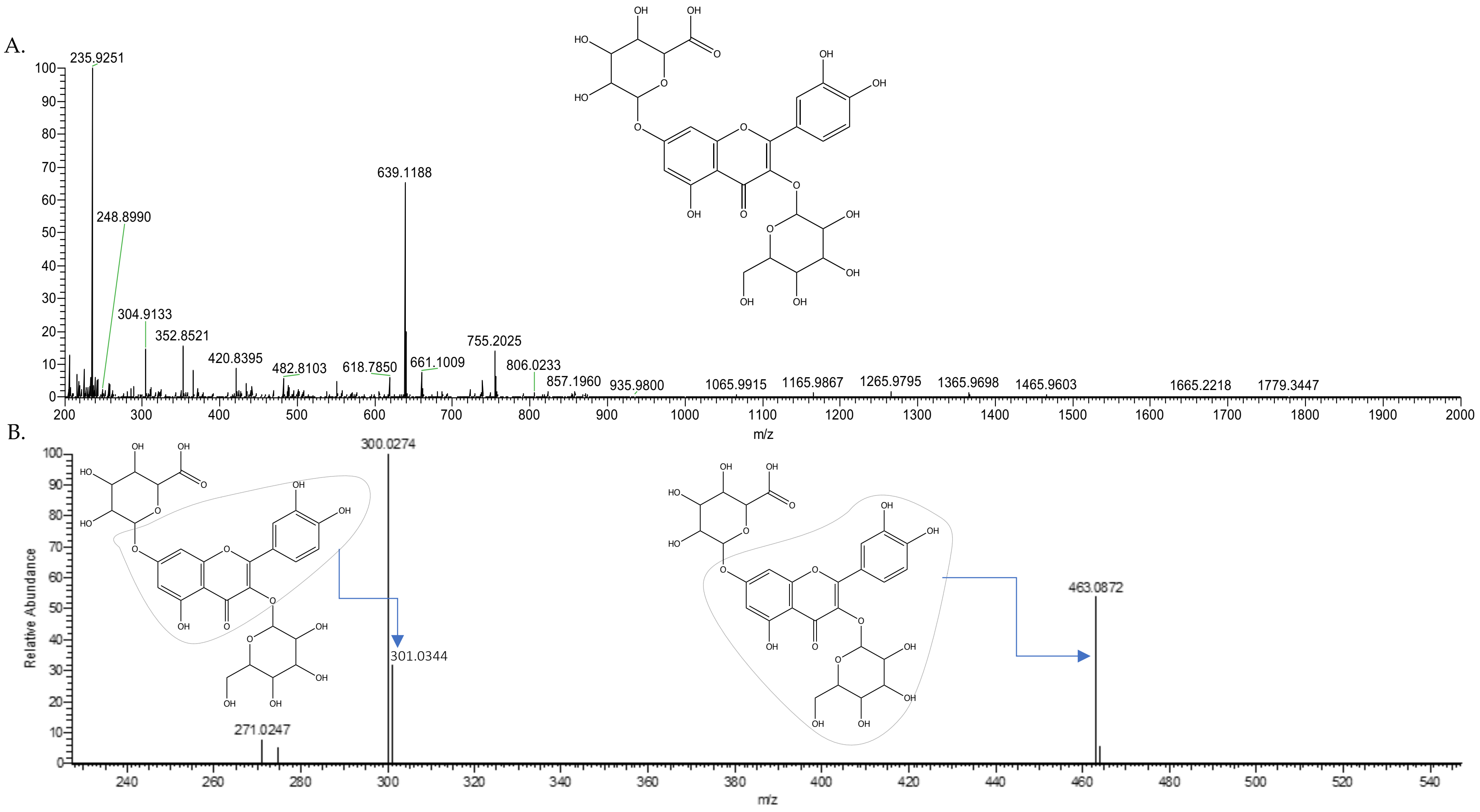

Figure S4 A) Full scan mass spectrometry (MS) spectrum corresponding to R_t 8.02 minutes. B) LC-MS/MS spectrum corresponding to fragmentation of m/z 639.1188, a quercetin hexoside glucuronide. Fragmentation patterns are indicated. Linkages of sugars to the aglycone backbone have not been determined, and only one orientation of the sugar linkages is shown for illustrative purposes.

molecules

MDPI

A.

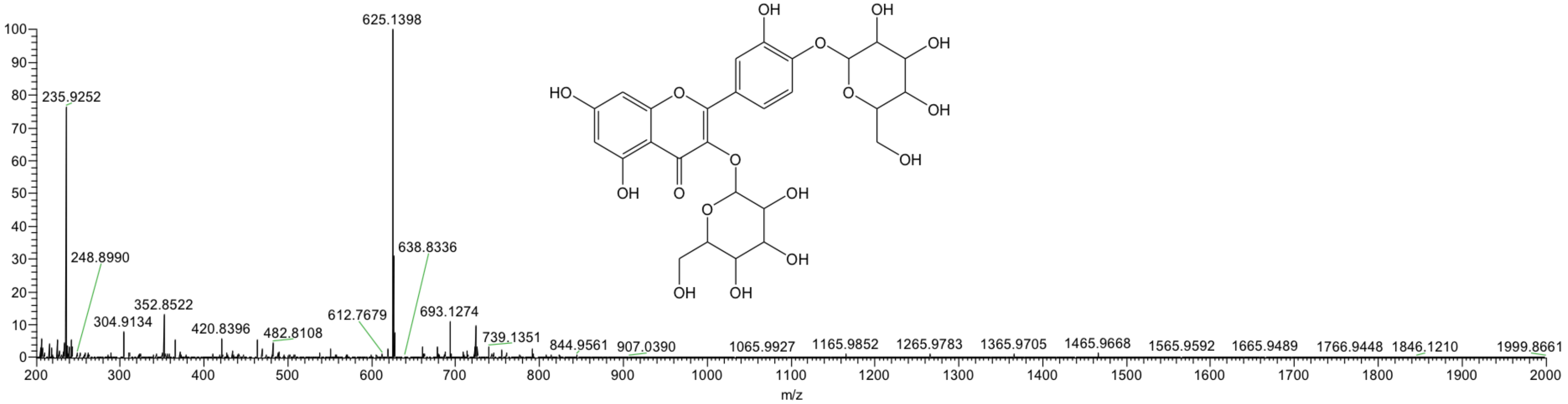

B.

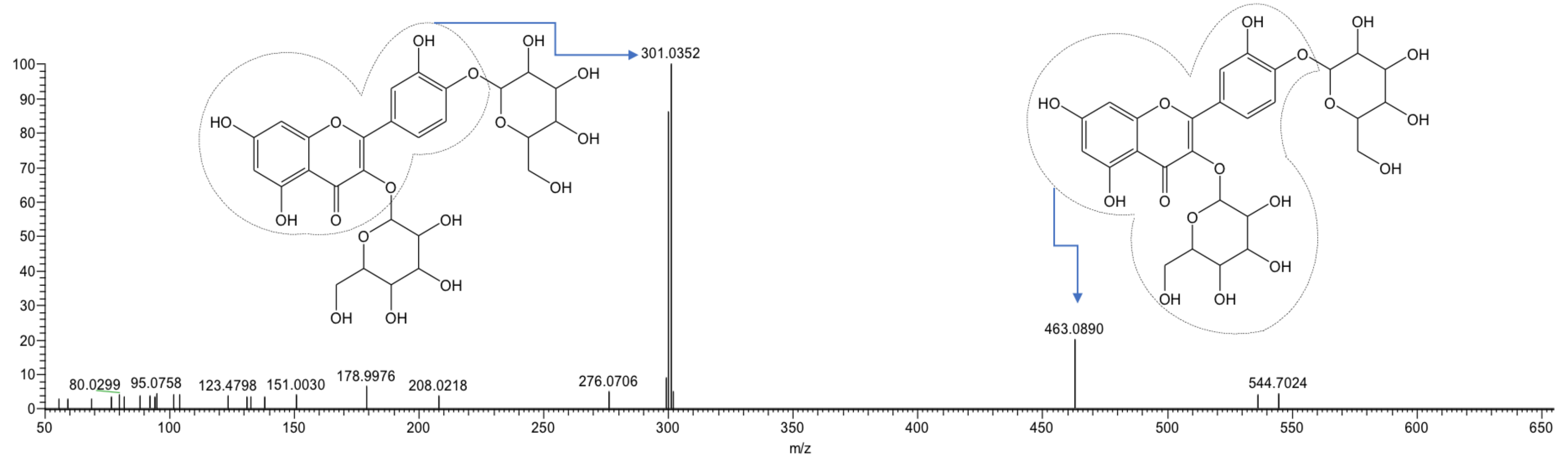

Figure S5 A) Full scan mass spectrometry (MS) spectrum corresponding chromatographic peak at R_t 12.65 minutes. B) LC-MS/MS fragmentation spectrum for m/z 625.1398, a quercetin sophoroside. Fragmentation patterns are indicated. Linkages of sugars to the aglycone backbone have not been determined, and only one orientation of the sugar linkages is shown for illustrative purposes.

molecules

MDPI

A.

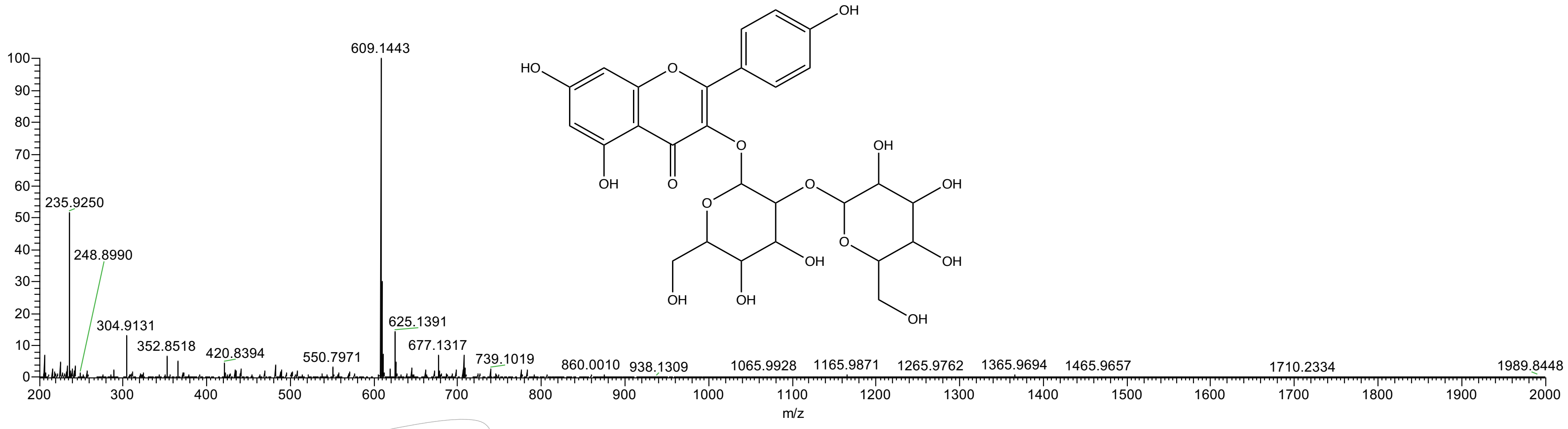

B.

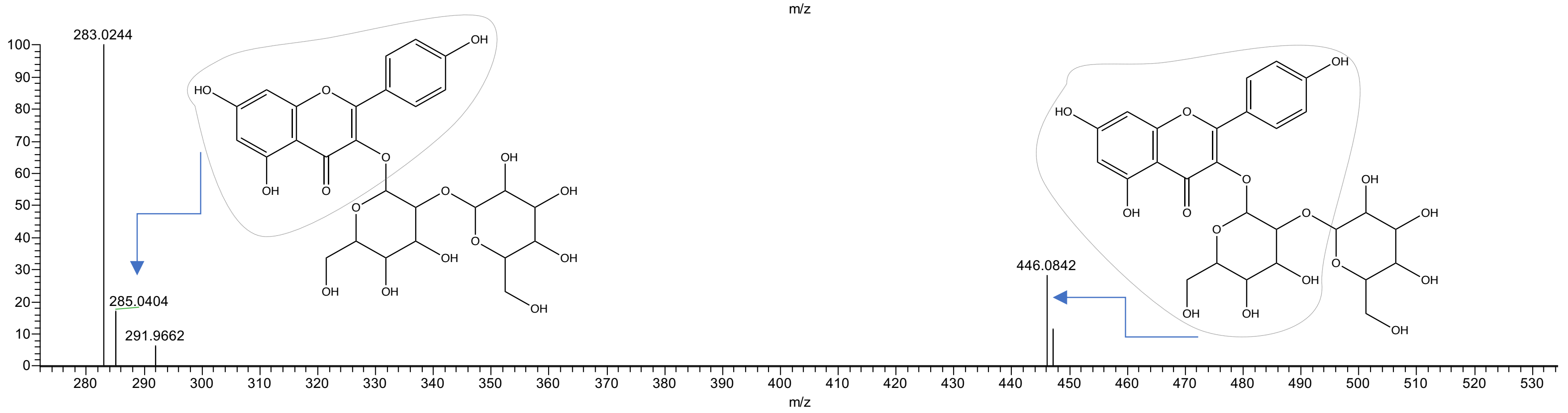

Figure S6 A) Full scan mass spectrometry (MS) spectrum corresponding chromatographic peak at R_t 12.72 minutes. B) LC-MS/MS fragmentation spectrum corresponding m/z 609.1443, a kaempferol sophoroside. Fragmentation patterns are indicated. Linkages of sugars to the aglycone backbone have not been determined, and only one orientation of the sugar linkages is shown for illustrative purposes.
